# Glycemic indices predict outcomes after aneurysmal subarachnoid hemorrhage: a retrospective single center comparative analysis

**DOI:** 10.1038/s41598-020-80513-9

**Published:** 2021-01-08

**Authors:** Matthew K. McIntyre, Mohamed Halabi, Boyi Li, Andrew Long, Alexander Van Hoof, Adil Afridi, Chirag Gandhi, Meic Schmidt, Chad Cole, Justin Santarelli, Fawaz Al-Mufti, Christian A. Bowers

**Affiliations:** 1 School of Medicine, New York Medical College, 40 Sunshine Cottage Rd, Valhalla, NY USA; 2 Department of Neurological Surgery, Oregon Health & Science University, 3181 SW Sam Jackson Park Road, Portland, OR USA; 3 Department of Neurosurgery, Westchester Medical Center, 100 Woods Rd, Valhalla, NY USA; 4 Department of Neurosurgery, University of New Mexico, 1 University of New Mexico, Albuquerque, NM 87131 USA

**Keywords:** Cerebrovascular disorders, Outcomes research

## Abstract

Although hyperglycemia is associated with worse outcomes after aneurysmal subarachnoid hemorrhage (aSAH), there is no consensus on the optimal glucose control metric, acceptable in-hospital glucose ranges, or suitable insulin regimens in this population. In this single-center retrospective cohort study of aSAH patients, admission glucose, and hospital glucose mean (MHG), minimum (MinG), maximum (MaxG), and variability were compared. Primary endpoints (mortality, complications, and vasospasm) were assessed using multivariate logistic regressions. Of the 217 patients included, complications occurred in 83 (38.2%), 124 (57.1%) had vasospasm, and 41 (18.9%) died. MHG was independently associated with (p < 0.001) mortality, MaxG (p = 0.017) with complications, and lower MinG (p = 0.015) with vasospasm. Patients with MHG ≥ 140 mg/dL had 10 × increased odds of death [odds ratio (OR) = 10.3; 95% CI 4.6–21.5; p < 0.0001] while those with MinG ≤ 90 mg/dL had nearly 2× increased odds of vasospasm (OR = 1.8; 95% CI 1.01–3.21; p = 0.0422). While inpatient insulin was associated with increased complications and provided no mortality benefit, among those with MHG ≥ 140 mg/dL insulin therapy resulted in lower mortality (OR = 0.3; 95% CI 0.1–0.9; p = 0.0358), but no increased complication risk. While elevated MHG and MaxG are highly associated with poorer outcomes after aSAH, lower MinG is associated with increased vasospasm risk. Future trials should consider initiating insulin therapy based on MHG rather than other hyperglycemia measures.

## Introduction

Despite advances in neurocritical care, aneurysmal subarachnoid hemorrhage (aSAH) remains devastating disease with an often unpredictable and complicated clinical course. Although historical grading systems, such as the Hunt and Hess (HH) and Fisher scores, continue to have clinical utility, higher hemorrhage grades do not universally foretell poorer outcomes and thus research into other potential prognostic variables is needed^1^.

Hyperglycemia after aSAH is well-described and is often attributed to an endogenous stress response after intracranial injury rather than the underlying insulin intolerance seen in diabetes^2^. Therefore, this physiologic response is considered both a potential biomarker and a treatment target. Years of study, however, have yielded a paucity of actionable results. For example, although hyperglycemia has been associated with worse hemorrhages^3,4^ and poorer outcomes after aSAH^3,5,6^, no consensus exists on which glycemic measures best predict aSAH outcomes. Meanwhile, multiple studies have demonstrated independent glucose effects, including glucose variability^7,8^, high admission day glucose (ADG)^2,3,9^, high mean hospital glucose (MHG)^5,10^, high maximum glucose (MaxG)^11^, and low minimum glucose (MinG)^12^, or no glucose effect^13^ on aSAH outcomes. Therefore, there is no consensus regarding acceptable in-hospital glucose ranges, appropriate cutoffs of the glycemic measures, or suitable insulin regimens in this population^14–16^. Without a consensus on the glucose measure that best predicts adverse outcomes in aSAH, it is challenging to determine optimal treatment paradigms for glycemic control following aSAH.

Vasospasm and other complications, such as pulmonary embolism, sepsis, pneumonia, and endovascular complications, are common among patients with aSAH and contribute to the high morbidity and mortality burden of this disease. Yet, there is a lack of reported data on glucose control’s association with these adverse events. The goals of this study are therefore to: (1) determine the optimal glycemic measure in aSAH for predicting mortality, complications, and vasospasm; (2) provide cutoff values that portend a potential risk of adverse events; and (3) examine insulin administration’s effects on mortality, complications, and vasospasm.

## Results

### Baseline features and demographics

Of the 251 identified patients, 217 met the study inclusion criteria, 13 were excluded for nonaneurysmal SAH, 14 for unavailable records, four for concurrent non-SAH neurosurgical or acute medical illness, and three were excluded for nonacute presentation of SAH. As expected, the majority were white (122/217, 56.2%) and female (142/217, 65.4%) (Table 1). The mean patient age was 57.6 ± 1.0 years. The average BMI across the cohort was 27.8 ± 0.5 kg/m^2^. The average HH score was 2.9 ± 0.1, and almost 1/3 of patients [66 (30.4%)] had high-grade aSAH (HH score 4 or 5). The most common aneurysm location was the anterior communicating artery (72/217, 33.2%), and the majority were treated with endovascular therapy (175/217, 80.6%). For other baseline characteristics, see Table 1.

**Table 1:** Baseline patient characteristics (n = 217).

Variable	Measurement^a^
Age (years)	57.6 ± 1.0
Female	142 (65.4%)
White	122 (56.2%)
Body mass index (kg/m^2^)^b^	27.8 ± 0.5
Anticoagulation/antiplatelet medication use	52 (24.0%)
**History of diabetes**	28 (12.9%)
Home metformin use	11 (5.1%)
Smoking history	87 (40.1%)
Hunt and Hess score	2.9 ± 0.09
Fisher score	3.7 ± 0.04
Aneurysm size (mm)^b^	6.0 ± 0.2
**Ruptured aneurysm location**	
Anterior cerebral (%)	9 (4.1%)
Anterior communicating (%)	72 (33.2%)
Basilar (%)	16 (7.4%)
Internal carotid (%)	23 (10.6%)
Middle cerebral (%)	31 (14.3%)
Posterior cerebral (%)	4 (1.8%)
Posterior communicating (%)	43 (19.8%)
Vertebral (%)	7 (3.2%)
Other (%)	14 (6.5%)
Mean admission A1c (n = 86)	5.7 ± 0.1
Mean ADG (mg/dL)	148.0 ± 3.6
Mean MHG	133.5 ± 2.1

^a^Measurement is reported as mean ± SEM or count (%).

^b^Non-normally distributed.

**Figure 1:**
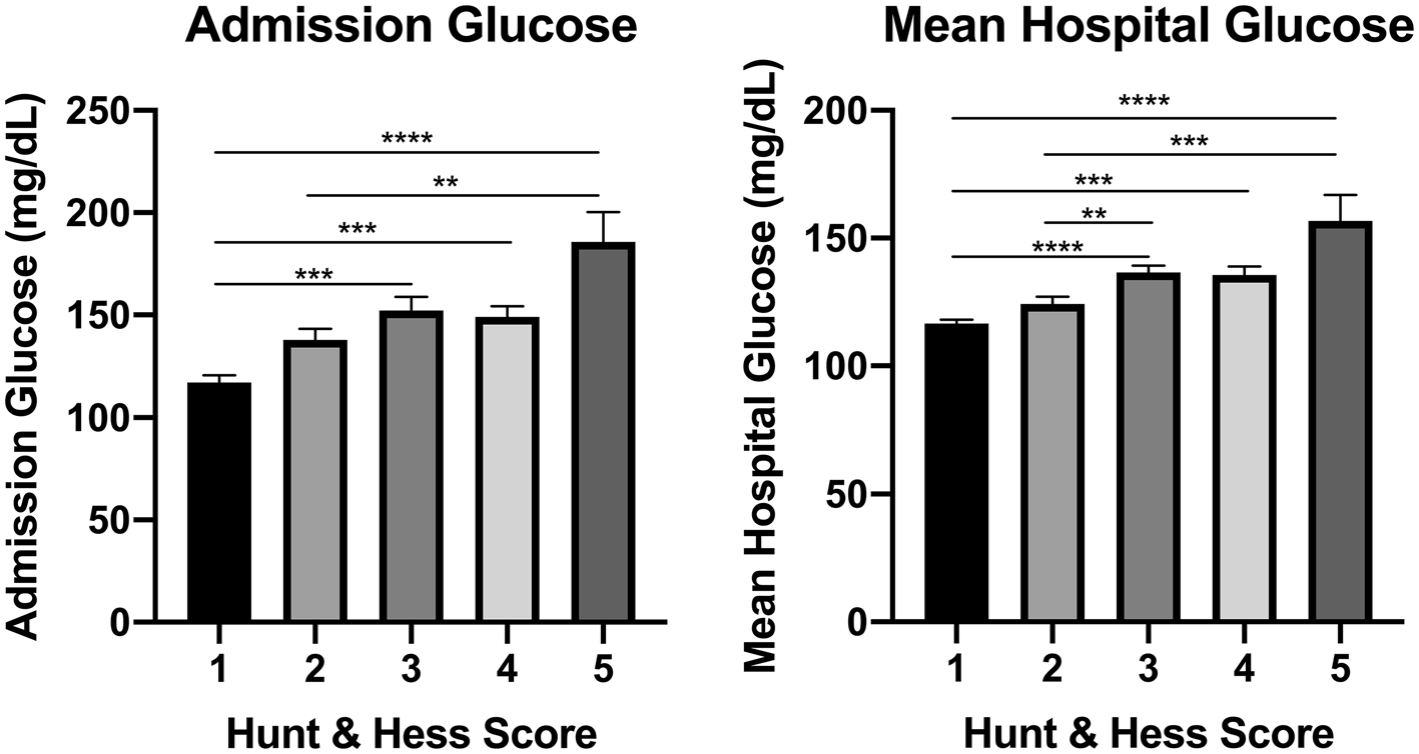
The effect of aneurysmal subarachnoid hemorrhage severity on mean admission day glucose (left) and mean hospital glucose (right). Asterisks indicate post-hoc significance among groups. **p < 0.01, ***p < 0.001, ****p < 0.0001.

Only 86 (39.6%) patients had an admission A1c, and 28 (12.9%) had prior diabetes diagnosis. A total of 79 (36.4%) patients received insulin during their admission, which was started, on average, 4.1 ± 0.6 days (range 0–32)] after admission. The average ADG was significantly higher than the MHG (148.0 ± 3.6 versus 133.5 ± 2.1; paired p < 0.0001), and both the ADG and the MHG were significantly related to increasing HH score in a grade-dependent manner (p < 0.0001, Fig. 1).

### Clinical course and outcomes

Among this cohort, 83 (38.2%) patients developed a complication, with pneumonia being the most common (56/217, 25.8%) followed by sepsis (19/217, 8.8%) (Table 2). Given the high rates and distinct aSAH-dependent cause of vasospasm (124/217, 57.1%), we did not include it in complication rate analysis but rather considered vasospasm as an independent primary endpoint. The average length of stay in the intensive care unit and hospital were 17.1 ± 0.7 and 20.8 ± 0.9 days, respectively. Forty-one (18.9%) patients died during their hospitalization. See Table 2 for the incidence of other secondary endpoints.

**Table 2:** Hospital course and clinical outcomes.

Variable	Measurement
Hospital LOS (days)	20.8 ± 0.9
ICU LOS (days)	17.1 ± 0.7
**Patients with complications**	83 (38.2)
Deep vein thrombosis	15 (6.9)
Pulmonary embolism	3 (1.4)
Sepsis	19 (8.8)
Pneumonia	56 (25.8)
Endovascular complication	11 (5.1)
Vasospasm	124 (57.1)
Mortality	41 (18.9)
Discharge home	74 (34.1)
Vasopressor use	85 (39.2)
Tracheostomy tube	48 (22.1)
Gastrostomy tube	53 (24.4)
Discharge GCS (without death)	14.1 ± 0.2 (range 6–15)
Extraventricular drain	164 (75.6)
Shunt-dependent hydrocephalus	18 (8.3)

Values are reported as mean ± SEM or number (percentage).

*LOS* length of stay, *ICU* intensive care unit, *GCS* Glasgow Coma Scale score.

aSAH patients who died were significantly older (p = 0.0001) and had lower BMI (p = 0.0063) and higher HH (p < 0.0001) and Fisher scores (p = 0.0009) compared with those who did not (Table 3). In terms of glycemic measures shown in Table 3, patients that died had higher ADG (p < 0.0001), MHG (p < 0.0001), standard deviation of glucose (p < 0.0001), MaxG (p < 0.0001), and MinG (p < 0.0001). Similarly, those who developed any complication had higher HH scores (p < 0.0001), higher ADG (p = 0.0418), MHG (p < 0.0001), standard deviation of glucose (p = 0.0028), and MaxG (p < 0.0001) and were more likely to have received insulin during their stay (p = 0.0136). Those who developed a complication also had lower admission A1c (p = 0.0241), but only a minority of patients (87/217, 40.1%) had this variable measured upon arrival and there were no differences in mortality, complication, or vasospasm rates among those with A1c ≥ 6.5%. Unlike the other primary endpoints, vasospasm was associated with younger age (p = 0.0099) and lower MinG (p = 0.0135).

**Table 3:** Factors associated with higher mortality, complications, and vasospasm.

Variables	Death			Complication			Vasospasm		
	No (n = 176)^a^	Yes (n = 41)^a^	p-value	No (n = 134)^a^	Yes (n = 83)^a^	p-value	No (n = 93)^a^	Yes (n = 124)^a^	p-value
Age (years)	55.8 ± 1.0	65.2 ± 2.5	**0.0001**	56.8 ± 1.2	58.8 ± 1.5	0.3152	60.4 ± 1.6	55.4 ± 1.2	**0.0099**
Female (%)	116 (65.9)	26 (63.4)	0.8555	87 (64.9)	55 (66.3)	0.8839	62 (66.7)	80 (64.5)	0.7744
White (%)	99 (56.3)	23 (56.1)	> 0.9999	72 (53.7)	50 (60.2)	0.3989	52 (54.8)	71 (57.3)	0.7826
BMI (kg/m^2^)^b^	28.4 ± 0.5	25.4 ± 0.7	**0.0063**	27.9 ± 0.6	27.7 ± 0.7	0.9331	27.1 ± 0.7	28.3 ± 0.6	0.0716
History of diabetes (%)	20 (11.4)	8 (19.1)	0.2007	16 (7.4)	12 (14.5)	0.6778	12 (12.9)	16 (12.9)	> 0.9999
HH score^b^	2.7 ± 0.1	4.0 ± 0.2	**< 0.0001**	2.6 ± 0.1	3.4 ± 0.1	**< 0.0001**	3.1 ± 0.1	2.8 ± 0.1	0.2120
Fisher score^b^	3.6 ± 0.1	4.0 ± 0.03	**0.0009**	3.6 ± 0.1	3.8 ± 0.05	0.1973	3.6 ± 0.1	3.7 ± 0.1	0.3208
ADG (mg/dL)^b^	138.3 ± 2.8	189.9 ± 13.4	**< 0.0001**	144.9 ± 5.0	153.1 ± 5.0	**0.0418**	151.9 ± 5.8	145.2 ± 4.6	0.3049
**Admission A1c**^**b,c**^	5.7 ± 0.1	5.6 ± 0.3	0.4172	5.8 ± 0.1	5.5 ± 0.2	**0.0241**	5.7 ± 0.2	5.7 ± 0.2	0.8670
> 6.5%	10 (13.9)	3 (21.4)	0.4369	9 (20.5)	4 (9.5)	0.2297	6 (16.2)	7 (14.3)	> 0.9999
**Glucose during hospital stay (mg/dL)**									
MHG^b^	126.4 ± 1.4	163.9 ± 7.8	**< 0.0001**	130.0 ± 3.1	139.1 ± 2.2	**< 0.0001**	136.5 ± 3.7	131.2 ± 2.4	0.2581
Individual standard deviation^b^	25.8 ± 1.2	34.5 ± 2.8	**< 0.0001**	25.0 ± 1.1	31.3 ± 2.2	**0.0028**	28.6 ± 2.0	26.5 ± 1.3	0.4737
MaxG^b^	221.0 ± 9.2	264.6 ± 16.9	**< 0.0001**	207.2 ± 6.5	264.9 ± 18.1	**< 0.0001**	234.7 ± 14.6	225.2 ± 9.3	0.5658
MinG^b^	79.4 ± 1.3	103.0 ± 8.5	**< 0.0001**	85.8 ± 2.9	80.7 ± 2.5	0.9996	90.6 ± 3.9	78.8 ± 1.9	**0.0135**
Insulin use during hospitalization (%)	59 (33.5)	20 (48.8)	0.0739	40 (29.9)	39 (47.0)	**0.0136**	30 (32.3)	49 (39.2)	0.3188

Boldface values are statistically significant.

^a^Values reported as mean ± SEM or count (%).

^b^Non-normally distributed.

^c^n = 87 patients.

### Multivariate regressions

Given the multiple univariate associations between glucose measures and primary endpoints, stepwise multivariate logistic regressions were performed to identify the independent risk factors (Table 4). We found that mortality was independently associated with greater age (OR = 1.04; 95% CI 1.02–1.08; p = 0.028), higher HH score (OR = 2.43; 95% CI 1.58–3.72; p < 0.001), and increased MHG (OR = 1.05; 95% CI 1.03–1.08; p < 0.001). Similarly, the incidence of hospital complications was also independently associated with higher HH score (OR = 1.53; 95% CI 1.19–1.92; p = 0.001) and increased MaxG (OR = 1.004; 95% CI 1.001–1.007; p = 0.017) but not age. Finally, we found that younger age (OR = 0.98; 95% CI 0.96–0.998; p = 0.031) and lower MinG (OR = 0.98; 95% CI 0.98–0.997; p = 0.015) were independently associated with vasospasm after aSAH. Fisher score, BMI, admission glucose, standard deviation of glucose, and insulin use during hospitalization were not independently associated with any primary endpoint.

**Table 4:** Multivariate analysis of primary endpoints.

Endpoint	Variables^a^	Multivariate OR (95% CI)	p-value
Mortality	Age	1.04 (1.02–1.08)	**0.028**
	Hunt and Hess score	2.43 (1.58–3.72)	**< 0.001**
	MHG	1.05 (1.03–1.08)	**< 0.001**
Complications	Age	1.00 (0.98–1.02)	0.997
	Hunt and Hess score	1.53 (1.19–1.96)	**0.001**
	MaxG	1.004 (1.001–1.007)	**0.017**
Vasospasm	Age	0.98 (0.96–0.998)	**0.031**
	MinG	0.98 (0.97–0.997)	**0.015**

Boldface values are statistically significant.

^a^Only variables included in final models are shown.

**Figure 2:**
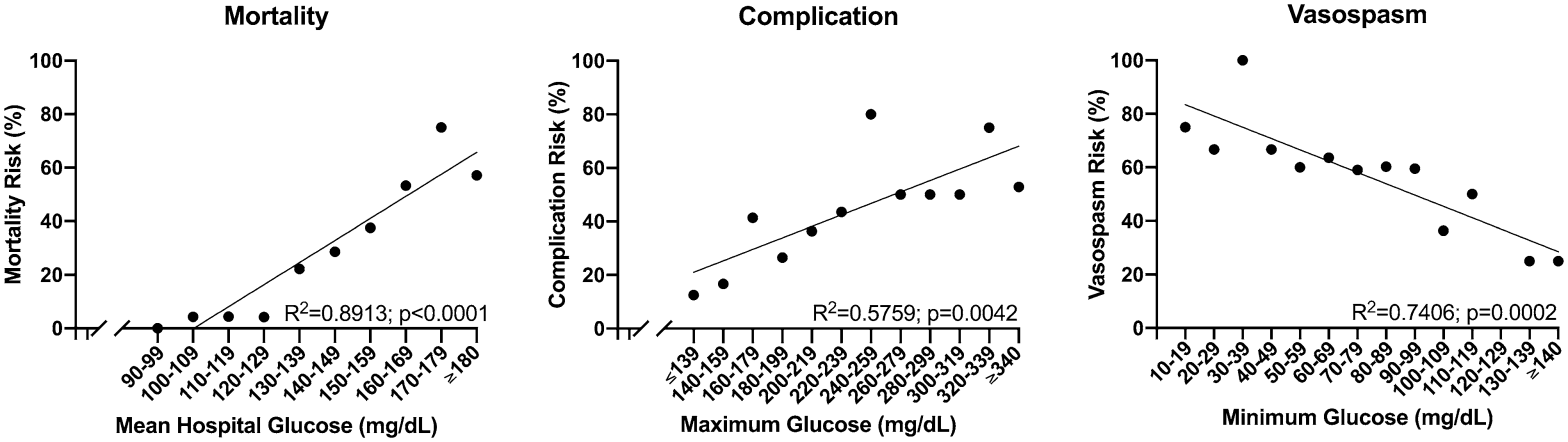
Risk of mortality (left), complications (middle), and vasospasm (right) versus glycemic variables that were significant in multivariate analysis. Each glycemic variable was binned into ordinal groups, and only groups with ≥ 2 members are shown. Simple linear regressions are shown.

To further explore the relationship between glucose measures and primary outcomes, variables that were significant in multivariate analysis were plotted against their respective endpoint (Fig. 2). This showed a significant dose–response relationship between MHG and mortality risk (R^2^ = 0.8913; p < 0.0001), between MaxG and complications (R^2^ = 0.5759; p = 0.0042), and between MinG and vasospasm (R^2^ = 0.7406; p = 0.0002).

### Direct comparison of glycemic indices for predicting primary endpoints

**Figure 3:**
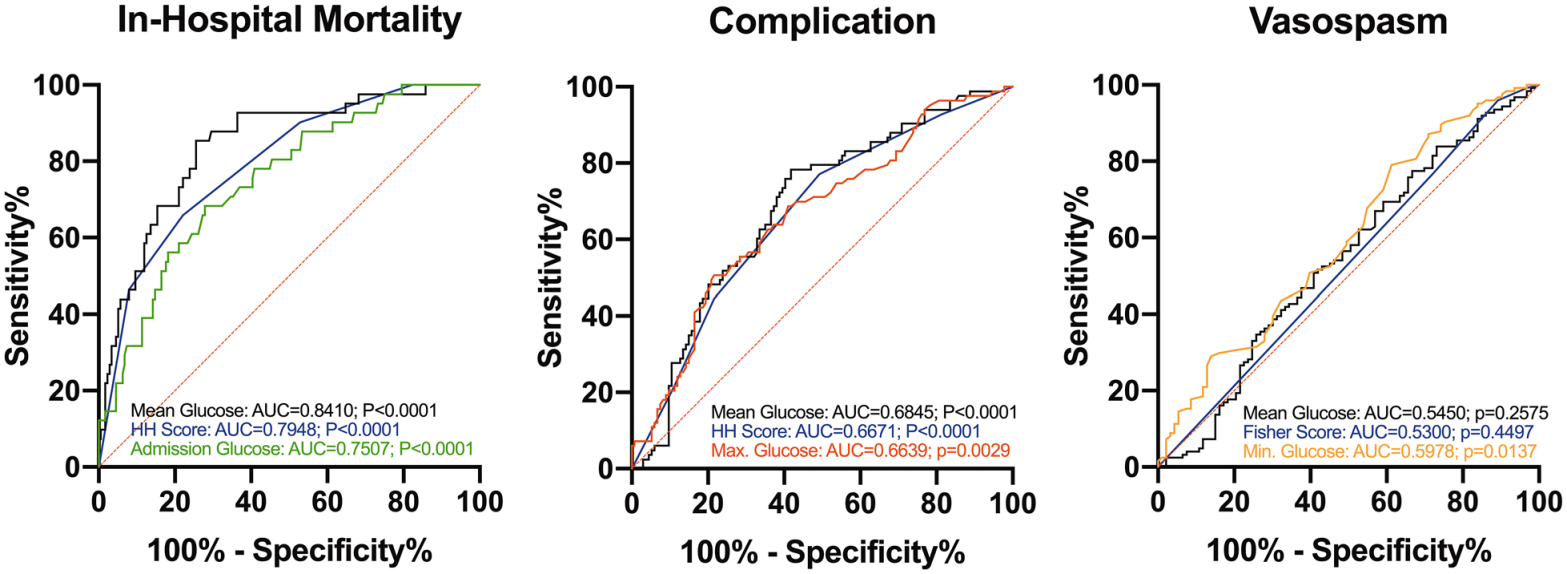
Receiver operating characteristic (ROC) curves for predicting in-hospital mortality (left), complications (middle), and vasospasm (right). Variables with corresponding area under the curve (AUC) are shown for the two glycemic variables with the highest significance. HH (left, middle) or Fisher score (right) are plotted for comparison.

Given the positive association between multiple glycemic indices and primary endpoints, ROC analysis was used to directly assess each variable and to determine an ideal cutoff for outcome prediction that could potentially be used clinically. As expected from multivariate analysis, MHG had the highest discriminatory value (AUC = 0.8410; p < 0.0001) for mortality, followed by HH score (AUC = 0.7948; p < 0.0001), and ADG (AUC = 0.7507; p < 0.0001) (Fig. 3, left). The standard deviation (AUC = 0.7403; p < 0.0001), MinG (AUC = 0.7309; p < 0.0001), and MaxG (AUC = 0.6974; p < 0.0001) also had significant, yet inferior, discriminatory ability for mortality.

Likewise, the MHG (AUC = 0.6845; p < 0.0001), followed by the HH Score (AUC = 0.6671; p < 0.0001), and MaxG (AUC = 0.6639; p = 0.0029) had the highest discriminatory value for complication development (Fig. 3, middle). ADG (AUC = 0.5822; p = 0.0419) and standard deviation of glucose (AUC = 0.6205; p = 0.0029) but not MinG (AUC = 0.5000; p = 0.9991) also had significant, but lower value, for complication development. In contrast, only MinG (AUC = 0.5978; p = 0.0137) had weak, but statistically significant, discriminatory value for vasospasm while HH score (AUC = 0.5482; p = 0.2245), MHG (AUC = 0.5450; p = 0.2575), MaxG (AUC = 0.5057; p = 0.8862), Fisher score (AUC = 0.5300; p = 0.4497), ADG (AUC = 0.5408; p = 0.3040), and standard deviation (AUC = 0.5285; p = 0.4723) did not (Fig. 3, right).

For ADG, a cutoff of 140 mg/dL was identified, and it was associated with 4.3× increased odds of mortality (OR = 4.3; 95% CI 2.1–9.5 p < 0.0001) but no difference in complication rate (OR = 1.1; 95% CI 0.7–2.0; p = 0.6741). Likewise, 140 mg/dL was the ideal cutoff for MHG for predicting mortality; patients with mean glucose ≥ 140 mg/dL had 10× higher odds of death (OR = 10.3; 95% CI 4.6–21.5; p < 0.0001) and a nearly 4× greater odds of a complication (OR = 3.7; 95% CI 2.0–6.6; p < 0.0001) compared with those with an MHG of < 140 mg/dL. The determined cutoff for MinG of ≤ 90 mg/dL was associated with higher rates of vasospasm (OR = 1.8; 95% CI 1.01–3.21; p = 0.0422) and decreased rates of mortality (OR = 0.2; 95% CI 0.1–0.3; p < 0.0001) but no complication rate differences (p > 0.05). One possible explanation for the finding that MinG ≤ 90 is associated with decreased mortality rates is that patients with a MinG of ≤ 90 mg/dL were 3× less likely to have a MHG of ≥ 140 mg/dL (OR = 0.30; 95% CI 0.2–0.6; p = 0.0002) and had a lower MaxG (p = 0.0258) than those with a MinG > 90 mg/dL. The cutoffs for admission and hospital glucose were not associated with differences in vasospasm rates (p > 0.05).

### Basis for insulin use: MHG, ADG, or history of diabetes?

Among the entire cohort, insulin use was associated with higher complication rates (OR = 2.1; 95% CI 1.2–3.6; p = 0.0136) and a trend toward an increased mortality (OR = 1.9; 95% CI 0.9–3.8; p = 0.0739), but no differences in vasospasm rates (OR = 1.4; 95% CI 0.8–2.4; p = 0.3188). Those that received insulin had a lower MinG than those who did not (75.8 ± 4.7 vs. 88.4 ± 1.6 mg/dl; p < 0.0001). Likewise, among those who received insulin and developed vasospasm, 78.3% received the first dose of insulin before they had documented vasospasm.

Using the aforementioned cutoffs, we sought to determine whether clinicians could use ADG or MHG to predict who would obtain a mortality benefit with inpatient insulin use (Table 5). Interestingly, among those who had high MHG (> 140 mg/dL), those who received insulin (47/68, 70.1%) had significantly lower mortality (OR = 0.3; 95% CI 0.1–0.9; p = 0.0358) and no increased complication risk (OR = 1.8; 95% CI 0.6–5.1; p = 0.4147) but increased vasospasm risk (OR = 5.3; 95% CI 1.7–15.2; p = 0.0067). Conversely, those who had high ADG and received insulin had no mortality benefit (OR = 1.1; 95% CI 0.4–2.6; p > 0.9999) or difference in vasospasm (OR = 1.8; 95% CI 0.8–3.9; p = 0.1636) but were 4× more likely to develop a complication (OR = 4.1; 95% CI 1.7–9.4; p = 0.0018). For comparison, among those with a history of diabetes, there was no effect of insulin administration on mortality, complication rate, or vasospasm (p > 0.05).

**Table 5:** The effect of insulin admission on rates of death, complications, and vasospasm.

Patient group	Insulin Treatment		Death		Complication		Vasospasm	
	No	Yes	OR (95% CI)	p-value	OR (95% CI)	p-value	OR (95% CI)	p-value
Entire cohort	138 (63.6%)	79 (36.4%)	1.9 (0.9–3.8)	0.0739	2.1 (1.2–3.6)	**0.0136**	1.4 (0.8–2.4)	0.3188
MHG ≥ 140 mg/dL	20 (29.9%)	47 (70.1%)	0.3 (0.1–0.9)	**0.0358**	1.8 (0.6–5.1)	0.4147	5.3 (1.7–15.2)	**0.0067**
ADG ≥ 140 mg/dL	50 (51.0%)	48 (49.0%)	1.1 (0.4–2.6)	> 0.9999	4.1 (1.7–9.4)	**0.0018**	1.8 (0.8–3.9)	0.1636
History of diabetes	6 (20.7%)	23 (79.3%)	0.2 (0.03–1.2)	0.1231	3.7 (0.4–48.4)	0.3553	7.5 (0.9–96.7)	0.1331

Bold values indicate statistical significance.

## Discussion

We present a single-center retrospective cohort study of 217 patients with aSAH that shows that elevated MHG and MaxG levels were independently associated with worse outcomes, and lower MinG was associated with vasospasm. Higher MHG was an independent risk factor for mortality and best discriminates for aSAH patients at risk of death. Higher maximum glucose was independently associated with complications, but MHG had the best discriminatory ability for this endpoint. Vasospasm was independently associated with *lower* MinG. Identifying these glycemic metrics that were independently associated with worse aSAH outcomes merits further prospective study in order to help with aSAH prognostication and to establish aSAH inpatient glucose management goals.

aSAH induces sympathetic nervous system activation, causing a cortisol and cytokine surge leading to hyperglycemia and insulin resistance^15,17^. Although there is a relationship between stress hyperglycemia during critical illness and subsequent diabetes risk^18^, the effect of prehemorrhage insulin resistance on post-aSAH hyperglycemia and aSAH outcomes is not clear. To this end, Beseoglu et al. investigated the effect of baseline glycated hemoglobin (A1c) on aSAH-induced delayed cerebral ischemia and 6-month outcomes, finding that A1c has no significant influence on either endpoint^2^. Conversely, Dumont et al. showed that younger age, female sex, modified Fisher score, history of diabetes, and alcohol abuse, but not hyperglycemia, were associated with symptomatic vasospasm, which itself was associated with higher mortality and poor outcomes^19^. We found that a diabetes history, BMI (with multivariate analysis), baseline A1c, or A1c ≥ 6.5% had no association with aSAH mortality, complications, or vasospasm risk (Table 3). These findings are consistent with previous work by Beseoglu et al. and suggests that stress-induced hyperglycemia has a far greater effect on aSAH outcomes than a history of diabetes before aSAH.

Despite the well-established relationship between hyperglycemia and noncranial complications among critically ill patients^20^, there is a surprising shortage of literature regarding this relationship in patients with aSAH. In one of the few studies examining this endpoint, Frontera et al. investigated the effect of average daily peak glucose > 105 mg/dL (aka glucose burden) on noncranial complications, including respiratory failure, sepsis, and heart failure, and cranial complications, such as aneurysm rebleeding, vasospasm, and infarction^11^. In multivariate analysis, mean peak glucose burden was associated with heart failure, respiratory failure, pneumonia, and brainstem herniation but no other cranial or noncranial complications. They found an association between higher mean glucose burden and increased mortality. Although this group used a slightly different glycemic index than those used in our study, their results mirror our own in showing an effect of high maximum and mean glucose on both noncranial complications and increased mortality.

Vasospasm is a common complication after aSAH and is a primary contributor to the disease’s high morbidity rate. The pathophysiology of vasospasm is not well understood and likely involves a complex interaction among blood products, vasoactive substances, and inflammatory cascades^21^. To date, there is conflicting evidence regarding the effect of glucose on vasospasm development, with previous literature reporting an association between both high^22,23^ and low^24^ glucose levels and subsequent vasospasm development. We found an independent (Table 4) and dose–response (Fig. 2) effect of MinG on vasospasm risk and a nearly twofold increased risk of vasospasm among those with a MinG ≤ 90 mg/dL. Interestingly, the patients with MinG ≤ 90 mg/dL also had a lower mortality rate, which is possibly explained by the decreased incidence of both high MHG and lower MaxG. This is similar to the findings of Naidech et al.*,* who showed that, lower MinG, but not MHG or MaxG, in patients with aSAH was associated with increased vasospasm and worse functional outcomes. They proposed a minimum threshold of 80 mg/dL for glucose control after aSAH. Together, these results suggest modern trials of insulin therapy in patients with aSAH may want to consider maintenance of MinG above 90 mg/dL to reduce vasospasm risk.

Insulin management for patients in the neurocritical care unit with aSAH is challenging as hypoglycemia, hyperglycemia, and intensive insulin therapy (targets 81–108 mg/dL) have all been associated with higher mortality and increased complications^25–27^. To date, only one small randomized trial in this population has compared intensive (target 80–120 mg/dL) versus conventional (target 80–220 mg/dL) insulin therapy, but its results were equivocal as there was no difference in vasospasm, mortality, and neurologic outcomes between the groups except for a lower infection rate with the intensive protocol^28^. The American Stroke Association guidelines on aSAH management only suggest “avoiding hypoglycemia,” without providing strict cutoff values defining hypoglycemia, and the European Stroke Organization suggests only treating blood glucose when ≥ 180 mg/dL^16,29^. Our study showed that, in general, insulin therapy did not improve mortality or vasospasm rates but was associated with greater complication risk. However, among the subset of patients with an MHG ≥ 140 mg/dL, insulin treatment was associated with a significantly lower mortality, no increased risk of complications, but a higher rate of vasospasm (Table 5). For comparison, we showed that among those who had an admission glucose ≥ 140 mg/dL, insulin use was again associated with an increased complication rate and no mortality benefit. Similarly, among those who had a history of diabetes, insulin use was not associated with any differences in primary endpoints. Together, these results suggest initiating insulin therapy among those with a mean hospital glucose ≥ 140 mg/dL, rather than based on a history of diabetes or high ADG, may be associated with better outcomes for these patients.

The principal limitation of this study is that it is a retrospective cohort study and thus carries all the intrinsic potential bias common with these studies. Another potential limitation is that we did not consider glucose measurements based on the day of hospitalization, time and dose of insulin administration, the relationship of insulin administration with food intake, and the temporal relationship between glucose changes and endponits. We also assessed glucose over the course of the entire hospital stay rather than day by day, because of the complexity and fluctuations throughout a normal day in critically ill aSAH patients, but this may bias our analysis of insulin administration and the predetermined cutoffs. Moreover, in our analysis it is challenging to discern causality between the abburations (high or low) in glucose and the examined endpoints. A prospective study that included these temporal relationships would help to reduce potential bias. A prospective study would also enable collection of other established predictors of vasospasm such as the Hijdra scale or hemorrhage volumetry in addition to further stratification of vasospasm into radiographic, clinical vasospasm resolved by augmenting the blood pressure and clinical vasospasm requiring intra-arterial vasodilator therapy delivered endovascularly. This type of study would also allow for collection of more granular datapoints such as delayed cerebral ischemia and for observation of long-term functional and mortality outcomes to provide more patient centered outcome analysis.

## Conclusion

In this retrospective study, we investigated several different glycemic metrics for use in patients aSAH, and we demonstrated that the MHG and MaxG were independently associated with mortality and complications, respectively, but that MinG was independently associated with increased vasospasm risk. We suggest parameters for future prospective trials with a target blood glucose range of 90–140 mg/dL to guide insulin therapy and that insulin initiation should be guided by the MHG, rather than admission glucose or a patient history of diabetes. Although prospective and randomized studies are needed, these retrospective and single center results provide additional evidence for the effect of both hyper- and hypoglycemia on outcomes after aSAH.

## Material and methods

### Study design and patient selection

This is a retrospective cohort study performed at Westchester Medical Center examining patients who presented with aSAH between June 2014 and July 2018. Institutional Review Board approval with a waiver of informed consent was obtained from New York Medical College and Westchester Medical Center. All research was performed in accordance with relevant guidelines and regulations.

Patients were identified by reviewing our institutional database of digital subtraction angiograms and selecting the patients with aSAH. Patients were excluded if they had nonaneurysmal or traumatic SAH, incomplete or unavailable records, concurrent non-SAH neurosurgical or acute medical illness, nonacute presentation of SAH, or lack of hemorrhage on imaging.

### Outcomes

Baseline information including patient demographics (age, sex, race), aneurysm size and location, Hunt & Hess (HH) and Fisher scores, admission Glasgow Coma Scale (GCS) score, anticoagulation/antiplatelet (AC/AP) medication use, body mass index (BMI), diabetic history, and smoking history were collected.

The following glycemic indices were collected: mean admission day glucose (ADG, all glucose measurements on the day of admission averaged), mean (MHG, all glucose measurements during the admission averaged), minimum (MinG, the minimum glucose measurement during the admission averaged), maximum (MaxG, the maximum glucose measurement during the admission averaged), and variability (as defined by individual standard deviation) of hospital glucose. Admission hemoglobin A1c and inpatient insulin usage were also collected. All glucose measurements in the medical record were considered irrespective of whether the value was obtained via serum or point-of-care testing and regarless of patient care level status. This assumes that if glucose was measured it was in line with the patients goals of care. The primary endpoints were complication development, angiographic vasospasm, and in-hospital mortality. A complication was defined as the presence of any of the following: deep vein thrombosis (DVT), pulmonary embolism (PE), sepsis, pneumonia, or endovascular complication. Angiographic vasospasm was defined as the documented presence of vasospasm in any imaging report during the stay including diagnostic/interventional angiograms, computed tomography angiography and/or magnetic resonance imaging angiography.

### Statistics

Statistical analysis was performed using Prism 8.3.1 (GraphPad Software Inc., La Jolla, CA) and SPSS version 26 (IBM Corp., Armonk, NY). Significance was defined as p < 0.05. Normal distributions were determined using an Anderson–Darling normality test. T-tests (paired or unpaired) and Mann–Whitney tests were used for parametric and nonparametrically distributed continuous samples with ≤ 2 groups, respectively. A Wilcoxon matched-paired signed rank test was used for nonparametric matched continuous variables for ≤ 2 groups. Analysis of variance (ANOVA) with Tukey post-hoc testing was used for normally distributed continuous variables with ≥ 3 groups, and Kruskal–Wallis tests with Dunn’s multiple comparisons were used if data were not normally distributed. Fisher’s exact tests were used for binary variables and corresponding odds ratios (OR) with 95% confidence intervals (95%CI) are shown. Continuous data were shown using mean ± standard error of the mean (SEM) while binary data are shown with the percentage.

Stepwise logistic regressions were performed for primary endpoints. Analyses included the following potential continuous variables using the forward conditional method: Fisher score, BMI, insulin use in the hospital, and the five glycemic indices listed above. In addition to stepwise analysis described above, for the endpoints of complications and mortality, regressions were adjusted for both age and HH score. For vasospasm analysis, curves were adjusted only for age given that, in both our study and in many others, only age but not HH score predicts vasospasm incidence. p < 0.01 was required for entry into models, and only significant variables in multivariate analysis are shown. Factors selected to be independent predictors of primary endpoints were then binned and plotted against risk of primary endpoint, and simple linear regressions were performed.

Receiver operating characteristic (ROC) curves were generated to determine the discriminatory value of glycemic indices for predicting primary endpoints. Area under the curve (AUC) was calculated for each variable with AUC = 1 being considered perfect discrimination and AUC = 0.5 being equal to chance. ROC significance was determined using the Wilson and Brown method, and optimal whole number cutoff was determined by the threshold at which sensitivity and specificity was maximized and rounding to the nearest whole number divisible by 10.
